# Comparison of Pharmaceutical Characteristics between Brand-Name Meropenem and Its Generics

**DOI:** 10.3390/antibiotics10091096

**Published:** 2021-09-10

**Authors:** Ping Yang, Shigeru Fujimura, Yawei Du, Bei Zhang, Li Yang, Masato Kawamura, Zhenhua Zhang, Suodi Zhai

**Affiliations:** 1 Department of Pharmacy, Peking University Third Hospital, Beijing 100191, China; guoguokate@163.com (P.Y.); duyawei2K@sina.com (Y.D.); zhangbei26@126.com (B.Z.); lilianyangli@163.com (L.Y.); 2 Division of Clinical Infectious Diseases & Chemotherapy, Tohoku Medical and Pharmaceutical University, Sendai 981-8558, Japan; m-kawamura@tohoku-mpu.ac.jp; 3 Department of Medical Affairs, Sumitomo Pharmaceuticals (Suzhou) Co. Ltd., Shanghai 200025, China; zhangzh@dsmpharm.com.cn

**Keywords:** meropenem, dissolution time, stability, morphology of particles, antimicrobial susceptibility patterns

## Abstract

This study aimed to provide comparative information of pharmaceutical properties, including particle morphology and distribution uniformity, solubility, presence of residual solvent and insoluble particles, and antimicrobial activities, between brand-name meropenem (Mepem^®^, BNM) and its six generic products (GPs A-F) marketed in China. Particles of GP-A and -C in dry powder had similar diameters of BNM, while other GPs were larger. Only BNM and GP-A were completely dissolved within 100 s in the lab condition. No insoluble particles >25 μm in diameter were detected in BNM and GP-E. Regarding stability of GPs solutions evaluated by concentration of open-ring metabolites at 6 h and 8 h, BNM showed the lowest open-ringed metabolite concentrates. Residual solvent of acetone detected in one GP showed the maximum value, while ethanol and ethyl acetate were detected both in product E and product F. The concordance rates (%) of minimum inhibitory concentration (MIC) of each generic compared to BNM were 89.5, 85, 87.5, 88, 88.5, and 86.5, respectively, although no significant difference was reached in MIC. Pharmaceutical characteristic differences between the BNM and GPs identified in this study could provide insights into understanding the deviations in the drug manufacturing processes of generic drugs.

## 1. Introduction

Generic drugs must contain the same quantitative and qualitative composition of active ingredients compared to their brand-name counterparts [1,2,3,4]. The prescription of generic drugs is an effective approach for reducing the cost [5,6] and increasing global access to first-line medication for the treatment of diverse diseases. In 2010, generic antibiotics represented more than two-thirds of the global consumption of medications [7]. Currently, generics are being used widely [8,9].

The marketing approval of the brand-name drugs requires the completion of Phase I–III clinical trials. The brand-name products might be required to conduct additional post-marketing studies [10] after they are on the market to test their safety and effectiveness in a large number of people. In order to achieve in vivo clinical equivalence, the quality of the brand-name drug and the generics should be consistent in vitro [11]. Based on the data of pharmaceutical consistency and bioequivalence (BE) studies, the generic products can be approved quickly. For drugs that are intravenously administered, only a pharmaceutical consistency test is required in most cases. Minor differences in ingredient composition and excipients might occur during manufacturing processes, and these minor differences might be tolerated in a consistency test.

Meropenem is one of the carbapenems often used in the treatment of severe infections [12,13,14], and for the treatment of infections due to drug-resistant *Pseudomonas aeruginosa* and *Enterobacteriaceae* [15,16,17,18]. Meropenem generics have been widely used across the world. Previous studies have characterized differences between brand-name meropenem and its generics marketed overseas in terms of pharmaceutical features as well as therapeutical efficacies [11,19,20,21,22]. Previous studies compared brand-name meropenem and its generics in Colombia and found that the therapeutic nonequivalence of meropenem products might be attributed to the different susceptibility to dihydropeptidase I (DHP-I) hydrolysis [20]. Studies conducted in Japan showed that brand-name meropenem and its generic products are different in dissolution time, which might be associated with the differences in the size of bulk particles and the amount of solubilizer [19]. In addition, it has been characterized that meropenem generics marketed in Europe had different dissolution times and stability compared to its originator product [11]. Studies conducted in India [22] described a method for the determination of relative potency of various generic brands of antibiotics and showed that in terms of active pharmaceutical ingredient, not all generics are inferior. Taken together, previous studies provided comparative data between brand-name meropenem and its generics marketed overseas. However, pharmaceutical differences between meropenem BNM and its generics manufactured and sold in China have not been reported.

The present study aimed to compare the differences in pharmaceutical characteristics with respect to morphology by scanning electron microscopy (SEM), dissolution time, insoluble particles, stability, residual solvent, and antibacterial activity between brand-name meropenem (BNM) (Mepem^®^) and its six major generic products in China.

## 2. Results

### 2.1. Particle Morphology Characterization under SEM

**Figure 1:**
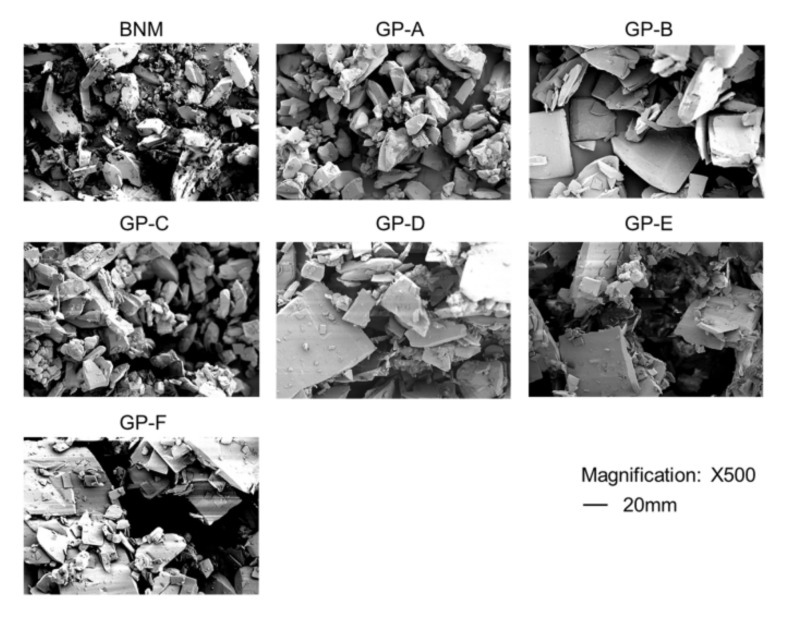
Images of particles of brand-name meropenem (BNM) and six generic products (GP-A to -F) using scanning electron microscopy. Magnification: 500×.

Results of the SEM of particles are displayed in Figure 1. The particle diameters of GP-A and -C were similar to that of BNM (about 40 nm), whereas the diameters of other GPs were greater than 80 nm. It can also be observed that meropenem particles of BNM, GP-A, and -C were evenly distributed and formed a more uniform mixture compared to other generics.

### 2.2. Dissolution Time Measurements

**Figure 2:**
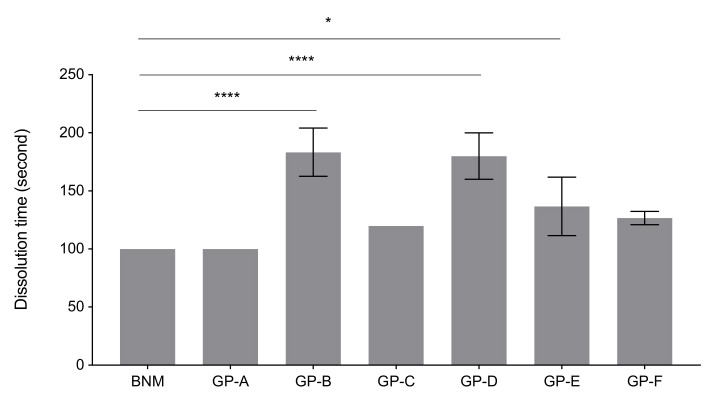
Comparison of dissolution time under laboratory conditions between BNM and six GPs (N = 3 batches. *p* < 0.05 was considered statistically significant. **** *p* < 0.0001, * *p* < 0.05).

Comparisons of the dissolution test of BNM and GPs under laboratory conditions are demonstrated in Figure 2. BNM and GP-A were completely dissolved within 100 s, while the others required at least 120 s. All three batches of GP-C solubilized completely within 120 s. Averaged time of complete dissolution of GP-B, -D, and -E were statistically longer than BNM, with GP-B (longer than 180 s) being the most difficult to dissolve. Besides, only one of the three batches of GP-F was completely dissolved within 120 s, while the other two batches required longer than 130 s.

**Figure 3:**
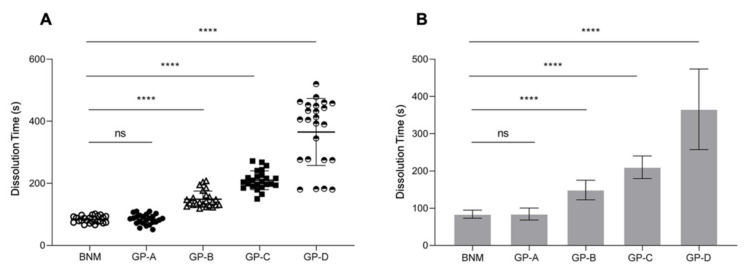
Comparison of dissolution time under simulated clinical conditions between BNM and six GPs (**A**): each dot represents one sample, 3 batches of samples were tested for each product, 8 samples tested for each batch; (**B**): data presented as mean ± standard deviation, ns: not significant (**** *p* < 0.0001).

Results of dissolution time measured under simulated clinical conditions are presented in Figure 3. The shortest mean dissolution time for BNM and GP-A was 84 ± 11 and 84 ± 16 s, respectively. On the other hand, GP-D needed a significantly longer time to dissolve (366 ± 108 s), while the mean dissolution time for GP-B and -C was 149 ± 26 and 210 ± 30 s, respectively. For inter-batch comparison, the visual dissolution time of GP-C and -D differed between the batches (*p* < 0.05). GP-E and -F came in containers that were smaller than the regular clinical size (10 mL). Therefore, comparison of dissolution time of GP-E and -F to BNM was not tested in this study.

### 2.3. Insoluble Particles Quantification

**Figure 4:**
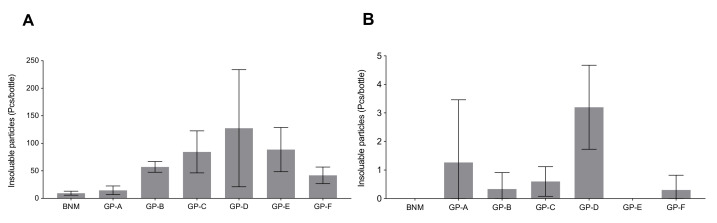
Number of insoluble particles detected in three batches of BNM and GPs (**A**): particle diameter greater than 10 μm, (**B**): particle diameter greater than 25 μm (N = 3 batches).

Comparisons of numbers of insoluble particles with diameters greater than 10 μm (Figure 4A) and 25 μm (Figure 4B) for BNM and GPs are presented in Figure 4. Among all the products tested, GP-D appeared to contain the highest number of insoluble particles larger than 10 μm in diameter, as well as more insoluble particles larger than 25 μm in diameter. No insoluble particles larger than 25 μm in diameter were observed in all three batches of BNM and GP-E.

### 2.4. Solution Stability

**Figure 5:**
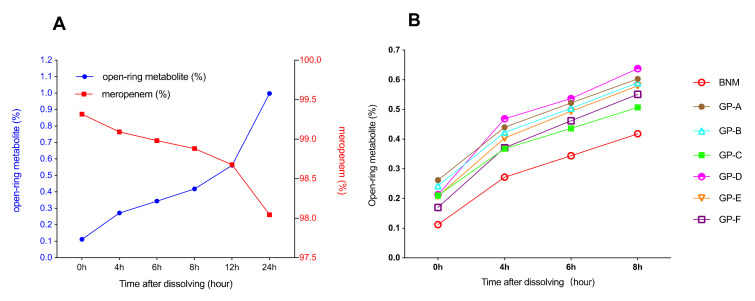
Comparisons of meropenem solution stabilities between BNM and GPs over time. (**A**) Changes in percentages of open-ring metabolites and remaining meropenem within the BNM solution over time. (**B**) Changes in open-ring metabolite concentrations within BNM and GPs solutions over time. Each line represents the average of three batches for each product.

The stability of the solution was evaluated by the percentage of open-ring metabolites of BNM and GP solutions over time. In order to prove that the percentage of open-ring metabolites in meropenem solution is representative of solution stability, we measured the percentage of meropenem remaining within the BNM solution over time and compared it with the percentage of the open-ring metabolites of BNM, which is showed in Figure 5A. It can be observed that the percentage of meropenem decreased while open-ring metabolites increased as a function of time, demonstrating the usefulness of open-ring metabolites in representing stability of meropenem solutions. The open-ring metabolites showed a tendency to increase over time for both the BNM solution and the GPs. GP-D and -A showed higher open-ringed metabolite concentrates over time, and GP-D demonstrated the fastest increase in metabolites from 0–4 h. At 4 h, all GPs showed comparable stability. At 6 h, GP-C, -E, and -F solutions showed lower concentrates of open-ring metabolite. At 6 h and 8 h, BNM showed the lowest open-ringed metabolite concentrates.

### 2.5. Residual Solvent Detection

**Figure 6:**
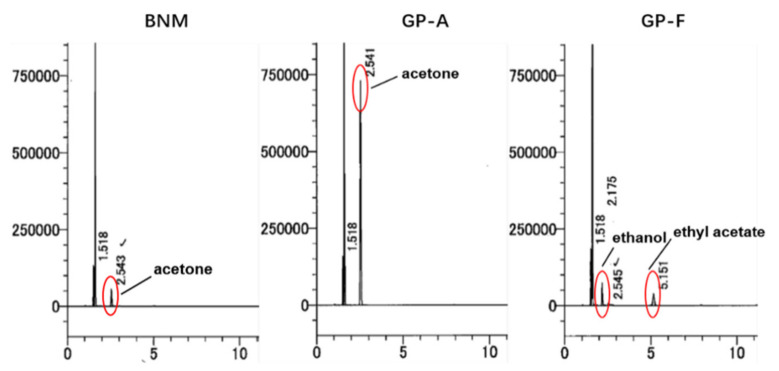
Chromatograms of the residual solvents in solutions of BNM (**left**) and GPs (**middle** and **right**). Red circles highlight peaks that indicate residual solvents (acetone, ethanol, and ethyl acetate) detected by headspace gas chromatography.

Residual solvent in meropenem solution was detected by gas-chromatography-mass spectrometry (GC-MS). For residual solvent, GP-A showed maximum acetone in all batches (0.23%), while ethanol and ethyl acetate were detected in both GP-E (0.09%, 0.01%) and GP-F (0.08%, 0.01%). (Figure 6)

### 2.6. Antimicrobial Susceptibility Test

Table 1 summarizes the results of antimicrobial susceptibility testing of BNM and six GPs against clinical isolates of *Pseudomonas aeruginosa* (*P. aeruginosa*). No significant differences were observed in the antimicrobial action between GPs and BNM. However, the MIC_88_ of GP-B, -D, and -F was higher than the other four products (8 µg/mL). The highest concordance rate was demonstrated by GP-A (89.5%) and -E (88.5%).

Concordance rate was calculated as the percentage of the GP’s MIC that was equal to one, two, four, and eight times BNM’s MIC.

## 3. Discussion

Differences between brand-name meropenem and meropenem generics marketed in foreign countries, including Japan [19], India [22], and European countries [11], have been reported by several studies [11,19,20,21,22]. This study, for the first time, characterized critical pharmaceutical features, including particle morphologies, solubility, numbers of insoluble particles, stability, residual solvents, and antimicrobial effects, of BNM and six major GPs marketed in China.

Although no statistically significant differences were observed in the HPLC quantification and the antimicrobial activity test, the BNM differs from the GPs in some pharmaceutical characteristics.

Previous studies characterized open-ring metabolites to be one of the major degradation products of meropenem, which are generated almost immediately after reconstitution [23,24]. Our results supported their findings by showing opposite trends of open-ring metabolites and meropenem concentrations in solution over time. The BNM solution remained stable for a little longer than GPs. The stability in the solution (reflected by open-ringed metabolites) was related to the quality of the formulation [25]. Meropenem with higher stability may be beneficial for clinical applications such as prolonged infusion. The instructions for meropenem recommended that the shelf-life of the saline-reconstituted solution to be 6 h before intravenous administration at room temperature. Therefore, prolonged infusion of meropenem solution during clinical applications should not be exceeding 6 h. Our results supported this finding: At 6 h post-reconstitution, BNM solution contained the minimum open-ring metabolites.

It has been shown by previous studies conducted in Japan that all eight meropenem generics marketed in Japan took a statistically longer time than brand-name meropenem to completely dissolve [19]. This study used similar methods to visualize meropenem particle morphologies of brand-name meropenem and its six generics under SEM and found that GP-A and -C were similar to the 40 nm of BNM, while for the other GPs, the diameter was >80 nm. A uniformity of mixing was observed in BNM.

Meropenem can be dissolved in 0.9% sodium chloride solution, 5% glucose solution, and glucose sodium chloride solution during intravenous application, in which the 0.9% sodium chloride solution is the most commonly used. Our study focused on the first and the most critical step of reconstitution in actual clinical applications—dissolving meropenem power in a small volume (usually 10 mL) of saline solution within its original vial. The difference in dissolution time might be related to the morphology of particles [26] (size and uniformity under SEM) and the uniformity in mixing. The particle size of the BNM was the smallest, which could arise due to variations in the recrystallization process [27] during manufacturing. In the current study, GP-B, -C, and -D required a long time to dissolve. One GP showed the same dissolution time of approximately 84 s as the BNM. In this study, the time of dissolution was subjectively and visually observed under simulated clinical conditions; the dissolution time was also tested under laboratory conditions.

In addition, according to the Chinese Pharmacopeia [28], insoluble particles for intravenous administration should not be exceeding 6000 for particles with diameters greater than or equal to 10 µm and 600 for particles with diameters greater than or equal to 25 µm per 1 g of sample. Our results indicated that while BNM and GPs both met the criteria of the Chinese Pharmacopeia, the number of insoluble particles in some GPs was larger than that in the BNM. According to the International Council for Harmonisation (ICH) of Technical Requirements for Pharmaceuticals for Human Use Guideline for Residual Solvents Q3C(R8) [29], acetone, ethanol, and ethyl acetate belong to Class 3 solvents, which are solvents with low toxic potential to humans. The limits of acetone, ethanol, and ethyl acetate in The Chinese Pharmacopoeia (2020) do not exceed 0.5%, which means that all products were in line with the limits of the Pharmacopoeia. The number of solvent residues between the BNM and GPs differed, which might be associated with the manufacturing technique and quality control of the generics.

*P. aeruginosa* has been shown to be one of the major causes of nosocomial infections, such as hospital-acquired pneumonia, sepsis, and bloodstream infections [30]. Treatment of *P. aeruginosa*-induced infections can be challenging due to its emergent resistance and limited choice of antibiotics [31]. *P. aeruginosa* isolates have been widely used to examine in vitro antimicrobial efficacy of meropenem [32,33]. Previous studies have examined MIC of brand-name meropenem and three generics marketed in Europe against *P. aeruginosa* isolates obtained from ICU and observed no statistical significances among these products, although some deviated results were observed [11]. Consistently, our study showed similar MIC against *P. aeruginosa* between BNM and six GPs, suggesting that generics showed reasonable equivalence with the BNM in terms of their antimicrobial activities, although the concordance rate was lower than 90%. Previous studies conducted in Japan have shown similar results [19].

Nevertheless, the present study has some limitations. The antimicrobial activity comparisons of BNM and GPs were only performed against *P. aeruginosa*. It is possible that significant differences in MIC against other bacteria strains may be detected between BNM and GPs. In addition, all the tests were conducted in vitro in laboratory conditions, and BE was not evaluated in animals or humans. Further clinical studies based on these findings may need to be implemented using brand-name meropenem and its generics.

## 4. Materials and Methods

### 4.1. Study Design and Subjects

The pharmaceutical characteristics of the BNM (Mepem^®^, Sumitomo Dainippon Pharma Co., Ltd., Osaka, Japan.) and its six major generic products (GPs) in China were assessed by SEM: dissolution time in both simulated clinical conditions and laboratory conditions, insoluble particles, solution stability, residual solvent, and antimicrobial susceptibility in three batches. The six GPs were represented by GP-A, -B, -C, -D, -E, and -F, respectively. The names of BNM and GPs were blinded to experimental personnel in all assessments.

### 4.2. Particle Morphology Characterization under SEM

Dry powder of BNM and each GP was examined using SEM to observe the morphology of the crystal particle [19] (Keyence, VE-8800, Osaka, Japan). The samples were added on a rectangular carbon tape attached to metal stubs; the excess powder was removed using compressed air. Then, the samples were placed in a sputter coater operated at 40 mA for 2 min (MSP-1S, Vacuum Device, Mito, Japan) for gold coating. The images were captured at 2 kV.

### 4.3. Dissolution Time Measurements

The dissolution tests were performed under both laboratory and simulated clinical conditions.

The dissolution time under laboratory condition was evaluated based on the time taken by 0.5 g BNM or GPs to dissolve in 5 mL normal saline at 25 °C with oscillation at 150 times/min on a constant temperature oscillator (SN200SD thermostatic water tank by Nissinrika, Tokyo, Japan and TRL-107NHF compact portable cooler by Thomas, Tokyo, Japan).

Dissolution time measured under simulated clinical conditions was recorded as the average of the observation by four pharmacists individually. Three batches of each product were tested. The pharmacists prepared BNM and its GPs in a clinical situation. Ten milliliters of saline were injected into the ampoule to dissolve 0.5 g BNM or GP-A, -B, -C, and -D. The dissolution was promoted by slight manual shaking. The time (s) required for the preparation of the solution to achieve complete dissolution of drug powder was observed by the naked eye and recorded. The average dissolution time was determined by the results obtained from four pharmacists for each batch of drugs and further compared to determine statistical significance.

In simulated clinical conditions, GP-E and -F were dissolved in 5 mL normal saline owing to the limitation of bottle size. This dissolution time may be different from that for those dissolved in 10-mL normal saline. Therefore, the dissolution time of GP-E and -F may be different from other products which were dissolved in 10-mL saline. Based on this consideration, data of dissolution time of GP-E and -F under simulated clinical condition was excluded from statistical analyses.

### 4.4. Insoluble Particles Quantification

Insoluble particles were detected by HIAC/ROYCO particles counter [34] (System 3000 A with a HRLD-150CE sensor) with the following parameters: flow velocity of 10 mL/min; the capacity and times of measurement were 5 mL × 4 times. Three batches were tested for each product. Numbers of particles with diameters larger than 10 µm and 25 µm were measured and analyzed in a respective manner.

### 4.5. Solution Stability

Meropenem solution stability test was performed using a HPLC system with a specialized UV detector (Shimadzu LC-20A, Kyoto, Japan). Instrument control, data acquisition and processing were controlled by LabSolutionsCS (ver. 6.84, Shimadzu). Reference meropenem standard was diluted in 0.1% triethylamine solution and used as an external standard to determine concentrations of meropenem and the open-ring metabolites in reconstituted solution (manufactured by Sumitomo Dainippon Pharma Co., Ltd., Oita, prepared by SCAS, Oita). Meropenem standard was diluted to 0.5 mg/mL to determine the concentration of remaining meropenem and 0.025 mg/mL to determine the concentration of the open-ring metabolites in solution, respectively. Each sample was diluted to 0.5 mg/mL in 0.1% triethylamine. Ten microliters of diluted sample were injected onto a liquid chromatography system (Column: 150 × 4.6 mm, 5 μm column filled with octadecylsilyl silica gel). The mobile phase was composed of 0.1% triethylamine and acetonitrile (93.5: 6.5, vol/vol) [28]. Meropenem retention time was 6.1 min, the open-ring metabolites retention time was 3.5 min. Wavelength of the UV detector was set to 220 nm and temperature of the column was set constantly to 25 °C. BNM and six GPs were tested at 0, 4, 6, and 8 h after dissolution in 10 mL normal saline at 25 °C.

### 4.6. Residual Solvent Detection

Residual solvent measurement of BNM and six GPs was performed using a qualified gas chromatography system (Shimadzu GC-2010 Plus) equipped with a headspace sampler (HS-20) following the instructions of the Chinese Pharmacopeia [28]. The headspace operating conditions are as follows: sample line temperature: 100 °C, transfer line temperature: 140 °C. Helium was used as the carrier gas at a constant flow rate. A capillary column (Column: 0.53 mm × 30 mm, 3.0 μm column coated with 100% dimethylpolysiloxane) of 40 °C was used to separate residual solvents from each sample in the gas chromatography system.

### 4.7. Antimicrobial Susceptibility Test

The antimicrobial susceptibility test was performed on a total of 200 non-duplicate clinical isolates of P. aeruginosa selected for this study and preserved on a bacterial bead (Microbank, IWAKI & Co., Ltd., Tokyo, Japan) at −80 °C at the Tohoku Medical and Pharmaceutical University. The minimum inhibitory concentration (MIC) was determined using the broth microdilution method [35,36] according to a standard procedure in accordance with the Clinical and Laboratory Standards Institute (CLSI) recommendations [37].

### 4.8. Statistical Analysis

Statistical analysis was conducted using SPSS Statistics for Windows, Version 17.0. The comparisons in the means and standard deviations of continuous variables with a normal distribution were performed using Student’s *t*-test. The median and range of continuous variables with a skewed distribution were compared using Wilcoxon rank-sum test. The *p*-values were based on two-sided tests and <0.05 was considered statistically significant.

## 5. Conclusions

In conclusion, although no statistically significant differences were observed in the HPLC quantification and the antimicrobial activity test, the studied meropenem generics were different from the brand-name meropenem in some pharmaceutical characteristics, such as stability, dissolution time, and morphology, as assessed by electron microscopy.

## Figures and Tables

**Table 1:** Antimicrobial susceptibility in vitro. MIC (µg/mL) and concordance rate (%) of each GP’s MIC to BNM against clinical isolates of *P. aeruginosa*.

	MIC (µg/mL)				Concordance Rate (%) of the MIC			
	MIC Range	MIC_50_	MIC_80_	MIC_88_	×1	×2	×4	×8
BNM	<0.6–32	0.5	2	4	n/a	n/a	n/a	n/a
GP-A	0.125–32	0.5	4	4	89.5	10.5	0	0
GP-B	0.125–32	0.5	2	8	85	14	1	0
GP-C	0.125–32	0.5	2	4	87.5	12	0.5	0
GP-D	0.125–32	0.5	2	8	88	11.5	0.5	0
GP-E	0.125–32	0.5	2	4	88.5	11	0.5	0
GP-F	0.125–32	0.5	2	8	86.5	12	1.5	0

MIC_50_, MIC_80_, MIC_88_ = MIC inhibits 50%, 80%, 88% of tested isolates. n/a = not applicable.

## Data Availability

The datasets used and/or analyzed during the current study are available from the corresponding author on reasonable request.
